# Two HCN4 Channels Play Functional Roles in the Zebrafish Heart

**DOI:** 10.3389/fphys.2022.901571

**Published:** 2022-06-30

**Authors:** Jiaying Liu, Go Kasuya, Buntaro Zempo, Koichi Nakajo

**Affiliations:** Division of Integrative Physiology, Department of Physiology, Jichi Medical University, Shimotsuke, Japan

**Keywords:** HCN4 channels, zebrafish, heart rate, morpholino, two-electrode voltage-clamp, ivabradine

## Abstract

The HCN4 channel is essential for heart rate regulation in vertebrates by generating pacemaker potentials in the sinoatrial node. HCN4 channel abnormality may cause bradycardia and sick sinus syndrome, making it an important target for clinical research and drug discovery. The zebrafish is a popular animal model for cardiovascular research. They are potentially suitable for studying inherited heart diseases, including cardiac arrhythmia. However, it has not been determined how similar the ion channels that underlie cardiac automaticity are in zebrafish and humans. In the case of HCN4, humans have one gene, whereas zebrafish have two ortholog genes (*DrHCN4* and *DrHCN4L*; ‘Dr’ referring to *Danio rerio*). However, it is not known whether the two HCN4 channels have different physiological functions and roles in heart rate regulation. In this study, we characterized the biophysical properties of the two zebrafish HCN4 channels in *Xenopus* oocytes and compared them to those of the human HCN4 channel. We found that they showed different gating properties: DrHCN4L currents showed faster activation kinetics and a more positively shifted G-V curve than did DrHCN4 and human HCN4 currents. We made chimeric channels of DrHCN4 and DrHCN4L and found that cytoplasmic domains were determinants for the faster activation and the positively shifted G-V relationship in DrHCN4L. The use of a dominant-negative HCN4 mutant confirmed that DrHCN4 and DrHCN4L can form a heteromultimeric channel in *Xenopus* oocytes. Next, we confirmed that both are sensitive to common HCN channel inhibitors/blockers including Cs^+^, ivabradine, and ZD7288. These HCN inhibitors successfully lowered zebrafish heart rate during early embryonic stages. Finally, we knocked down the HCN4 genes using antisense morpholino and found that knocking down either or both of the HCN4 channels caused a temporal decrease in heart rate and tended to cause pericardial edema. These findings suggest that both DrHCN4 and DrHCN4L play a significant role in zebrafish heart rate regulation.

## Introduction

The heart, an organ that pumps blood throughout the body, uses action potentials to contract the atria and ventricles regularly. A region called the sinoatrial node acts as a “pacemaker” by spontaneously generating its action potentials, leading to atria and ventricle action potentials. In pacemaker cells of the sinoatrial node that generate spontaneous action potentials, the pacemaker current or the funny current (I_f_) is activated by hyperpolarization (Brown and Difrancesco, 1980; Yanagihara and Irisawa, 1980; Baruscotti et al., 2005). Its molecular entity is a hyperpolarization-activated cyclic nucleotide-gated (HCN) channel. Therefore, HCN channels are also known as pacemaker channels because they control heart rate by generating pacemaker potentials (Ludwig et al., 1999; Moosmang et al., 2001; Brioschi et al., 2009). In addition to the heart, HCN channels are expressed in the central nervous system (Santoro et al., 2000) and the gastrointestinal nervous system (Fujii et al., 2020), where they play a role in the regulation of neuronal excitability and synaptic transmission (Beaumont and Zucker, 2000).

There are four known HCN genes in humans and other mammals: HCN1, HCN2, HCN3, and HCN4. All of the HCN channels are voltage-gated channels activated by membrane hyperpolarization (Ludwig et al., 1998). Each gene encodes a six-transmembrane α subunit, and four subunits combine to form a single HCN channel. The intracellular region contains a cyclic nucleotide-binding domain (CNBD), where cyclic nucleotides bind. Cyclic nucleotide (primarily cAMP in physiological condition) binding can facilitate activation and shift the voltage dependence of the HCN channels. For the HCN2 and HCN4 channels, the cAMP binding is thought to be an important molecular mechanism by which sympathetic activation increases heart rate. Furthermore, the HCN4 channel is mainly expressed in the heart, and mutations in the HCN4 gene may cause sick sinus syndrome and other cardiac disorders (Milanesi et al., 2006; Verkerk and Wilders, 2015). It has also been reported that knockdown of HCN4 channels in mice leads to bradycardia and cardiac block (Herrmann et al., 2007; Baruscotti et al., 2011).

There are some common inhibitors/blockers available for HCN channels including ivabradine (Bucchi et al., 2006, 2002), ZD7288 (BoSmith et al., 1993), and Cs^+^ (Sartiani et al., 2017). In a previous study using rabbits, it was shown that ivabradine can reduce heart rate by inhibiting pacemaker current I_f_ (Bucchi et al., 2002). In a clinical setting, ivabradine has been approved for treatment of chronic heart failure and reduction of heart rate. It has been reported that administration of ivabradine to patients reduces heart rate by about 20% (Swedberg et al., 2010).

The zebrafish (*Danio rerio*) is a small freshwater fish that is native to India, and it has a body length of 3–5 cm as an adult. It is easy to rear, has rapid embryonic development, and is highly fertile. The body is transparent for about 1 week after fertilization, allowing direct observation of tissues and organs. In addition, zebrafish are vertebrates having organs like humans and are frequently used as model animals in various fields including genetics and developmental biology. Transparency in the early developmental stages has attracted attention in the fields of cardiovascular research, toxicology, and drug discovery because it is possible to directly observe beating hearts (Milan et al., 2006; MacRae and Peterson, 2015; Narumanchi et al., 2021; Maciag et al., 2022). In zebrafish, ionic currents that underlie cardiac automaticity and excitability have also been studied in detail (Nemtsas et al., 2010; Vornanen and Hassinen, 2016; Ravens, 2018; Stoyek and Quinn, 2018). While most ion channel genes are common in humans and zebrafish, different ion channel genes are sometimes used in zebrafish hearts; for example, *KCNH6*, in addition to *KCNH2*, underlies a hERG like current in zebrafish (Vornanen and Hassinen, 2016). In addition, due to the genome duplication in teleost evolution, the zebrafish genome sometimes has two orthologs to one gene in mammals (Amores et al., 1998; Glasauer and Neuhauss, 2014). In the case of HCN4, there is only one HCN4 gene in the human genome, HsHCN4 (Hs: *Homo sapiens*), whereas the zebrafish genome contains two HCN4 channel genes, DrHCN4 (Dr: *Danio rerio*) and DrHCN4L (Fujii et al., 2020; von der Heyde et al., 2020). It has been shown that DrHCN4 is expressed in the heart and gastrointestinal tract (Fujii et al., 2020; Stoyek et al., 2022, 2015). Cardiac automaticity in zebrafish utilizes membrane mechanism along with calcium clock mechanisms as in mammals (Marchant and Farrell, 2019; Stoyek et al., 2022). In zebrafish, HCN4 is one of the key components in the membrane clock mechanisms as well (Stoyek et al., 2022, 2015). However, there have been no reports on the biophysical, pharmacological, and functional differences of these two zebrafish HCN4 channels.

In this study, we expressed these two zebrafish HCN4 channels and human HCN4 channels in *Xenopus* oocytes and compared their biophysical and pharmacological properties. We also studied the physiological role of these two zebrafish HCN4 channels in developing zebrafish embryos by administering inhibitors and knocking down the gene with antisense morpholino.

## Materials and Methods

### Reverse Transcription-PCR

Total RNA was extracted and purified from zebrafish larvae on days 1, 3, and 7 and from the heart and brain of adult fish using NucleoSpin RNA Plus (Takara Bio, Shiga, Japan). Reverse transcription was performed using PrimeScript™ RT Master Mix (Takara Bio). The obtained cDNA was stored at −20°C and later used for PCR as a template. Partial cDNA fragments of DrHCN4, DrHCN4L, and β-actin (control) were amplified using the primers listed below.

DrHCN4-Fw: AGT​GGA​CAA​CTT​CAA​CGA​GGT​GCT​G.

DrHCN4-Rv: AGG​GAG​CCC​GTT​TGA​GGG​TGT​TTT​GGT​GG.

DrHCN4L-Fw: TGT​GGA​CCA​TTT​TAA​CGA​GGT​GTT​GGA​GG.

DrHCN4L-Rv: GGA​TGC​GAA​CTG​TAG​GGA​GCC​CGT​TTG​AGG​GTG.

β-actin-Fw: TAA​TAC​ACA​GCC​ATG​GAT​GAG​G.

β-actin-Rv: GGG​AGC​AAT​GAT​CTT​GAT​CTT​C.

### Preparation of cRNA for Expression in *Xenopus laevis* Oocytes

The zebrafish HCN4 genes DrHCN4 (NCBI Reference sequence number: XM_680986.7) and DrHCN4L (XM_679638.8) were synthesized by GenScript (Piscataway, NJ, United States). They were amplified by PCR using primers containing the Kozak sequence (GCCACC) near the start codon and subcloned into a *Xenopus laevis* oocyte expression vector (pGEMHE) (Liman et al., 1992) using the In-Fusion HD Cloning Kit (Takara Bio). The human HCN4 gene, HsHCN4 (NP_005468.1), was purchased from Promega (Madison, WI, United States) and amplified with primers containing the N-terminal EcoRI site with the Kozak sequence and the C-terminal HindIII site. The PCR fragments were subcloned between the EcoRI and HindIII sites of the pGEMHE vector. These constructs were used as templates for cRNA synthesis after linearization with NheI. cRNA was synthesized using the mMESSAGE mMACHINE™ T7 Transcription Kit (Thermo Fisher Scientific, Waltham, MA, United States).

### *Xenopus laevis* Oocyte Injection

Female frogs (*Xenopus laevis*) were anesthetized in water containing 0.1% tricaine (Sigma-Aldrich, St. Louis, MO, United States, E10521), and oocytes were surgically obtained. To remove the follicular cell layer, oocytes were treated with 2 mg/ml collagenase (Sigma-Aldrich, C0130) dissolved in MBSH solution (88 mM NaCl, 1 mM KCl, 2.4 mM NaHCO_3_, 10 mM HEPES, 0.3 mM Ca(NO_3_)_2_, 0.41 mM CaCl_2_, 0.82 mM MgSO_4_, pH 7.6) for 6 h at room temperature. Defolliculated oocytes from stages V to VI were selected for cRNA injection. The synthetic cRNA (1–10 ng) was injected into each oocyte using Nanoject II (Drummond Scientific Company, Broomall, PA, United States). cRNA-injected oocytes were incubated in MBSH solution supplemented with 0.1% penicillin-streptomycin (Sigma-Aldrich, P4333) at 18°C for 2–3 days. All animal experiments using *Xenopus laevis* were approved by the Animal Experiment Committee of Jichi Medical University (Approval No. 18027).

### Two-Electrode Voltage Clamp

Ionic currents were measured by using an OC-725C amplifier (Warner Instruments, Hamden, CT, United States) with a two-electrode voltage clamp. Generation of voltage-clamp protocols and data acquisition were performed using a Digidata 1550 interface (Molecular Devices, San Jose, CA, United States) controlled by Clampex 10.7 software (Molecular Devices). Data were sampled at 10 kHz and low-pass filtered at 1 kHz using Clampex 10.7 software (Molecular Devices). All experiments were performed at room temperature. A glass electrode with a resistance of 0.2–0.5 MΩ was prepared from a borosilicate glass capillary (GC150TF-10, Harvard Apparatus, Holliston, MA, United States) using a micropipette puller (P-1000, Sutter Instrument, Novato, CA, United States). The glass electrode was filled with 3 M KCl. ND66 solution (66 mM NaCl, 32 mM KCl, 1 mM MgCl_2_, 1.8 mM CaCl_2_, 5 mM HEPES, pH 7.6) was used as the extracellular solution (Decher et al., 2003; Liu et al., 2020).

### Data Analysis

Activation curves (G-V curves) obtained by plotting the tail current at -120 mV were fitted using Clampfit 10.7 software (Molecular Devices) to a single Boltzmann function.

$$\text{G}={\text{G}}_{\min}+\left({\text{G}}_{\max}-{\text{G}}_{\min}\right)/\left(1+{\text{e}}^{-\frac{\text{zF}\left(\text{V}-{\text{V}}_{\frac{1}{2}}\right)}{\text{RT}}}\right).$$

where G_min_ is minimum tail current, G_max_ is maximum tail current, z is effective charge, V_1/2_ is half activation potential, F is Faraday constant, R is gas constant, T is absolute temperature.

To analyze the rate of activation, a current of -130 mV was fitted to a single exponential function (equation below) using pClamp 10.7 software to obtain the time constant (τ).

$$\text{I}\left(\text{t}\right)=Ae^{-\;\frac{t}{\tau }}+C$$

### HCN Channel Inhibitors

The HCN inhibitors ZD7288 (Z3777) and ivabradine hydrochloride (SML 0281) were purchased from Sigma-Aldrich. ZD7288 and ivabradine were dissolved in distilled water, and stock solutions of 100 mM or 50 mM were stored at −20°C. Cesium chloride was dissolved in distilled water, and 1 M of the solution was stored at room temperature. The stock solution was diluted with ND66 solution to achieve the final concentration on the day of the experiment.

For zebrafish, the HCN inhibitors at a final concentration of 0.01–1 mM were applied to egg water in which the fish were kept, starting at 24 h post-fertilization (hpf). Heart rates were recorded before (0 h) and at 24 h (24 h) and 48 h (48 h) after the inhibitors had been applied.

### Fish Maintenance and Egg Collection

Wild-type zebrafish (RIKEN-WT) were used. Zebrafish were kept in water at 28°C with 14 h of light (8:00–22:00) and 10 h of darkness (22:00–8:00). On the day before spawning, male and female fish were placed in a mating tank separated by a partition. On the morning of the spawning day, the partition was removed. Immediately after spawning, eggs were collected in a 10 cm dish with egg water (0.006% sea salt (Aquarium Systems, Sarrebourg, France) and 0.01% methylene blue). Animal experiments using zebrafish were approved by the Animal Experiment Committee of Jichi Medical University (Approval No. 18037).

### Heart Rate Measurement

The heart rate of zebrafish embryos was measured under an Olympus MVX10 microscope at room temperature. After zebrafish embryos were acclimated to the new environment (room temperature, light), heart rate was counted by eye.

### Gene Knockdown by Morpholinos

Morpholino antisense oligos (MO) were purchased from Gene Tools (Philomath, OR, United States) (Fujii et al., 2020). Zebrafish 1-cell embryos were injected with approximately 5 nL of MO solution at a concentration of 200 μM (approximately 10 ng/embryo).

The MO sequences used are listed below.

Negative Control (5′-CCT​CTT​ACC​TCA​GTT​ACA​ATT​TAT​A -3′).

DrHCN4 (5′-GTA​ATT​ACT​GCC​ACC​GTG​CAC​CAC​A-3′).

DrHCN4L (5′-GGC​GAC​GCT​GGC​TGA​AAA​ATA​GGT​C -3′).

### Statistical Analysis

Student’s *t*-tests were used to compare two groups, and the Dunnett or Tukey multiple comparison tests were used for multiple comparisons using EZR software (EZR version 1.54) (Kanda, 2013). *p* < 0.05 was considered as a significant difference. *p* < 0.05, <0.01, and <0.001 are denoted in the figures by *, **, and ***, respectively. All exact *p* values are listed in the tables.

## Results

### DrHCN4 and DrHCN4L Encode Functional Hyperpolarization-Activated Channels

**FIGURE 1:**
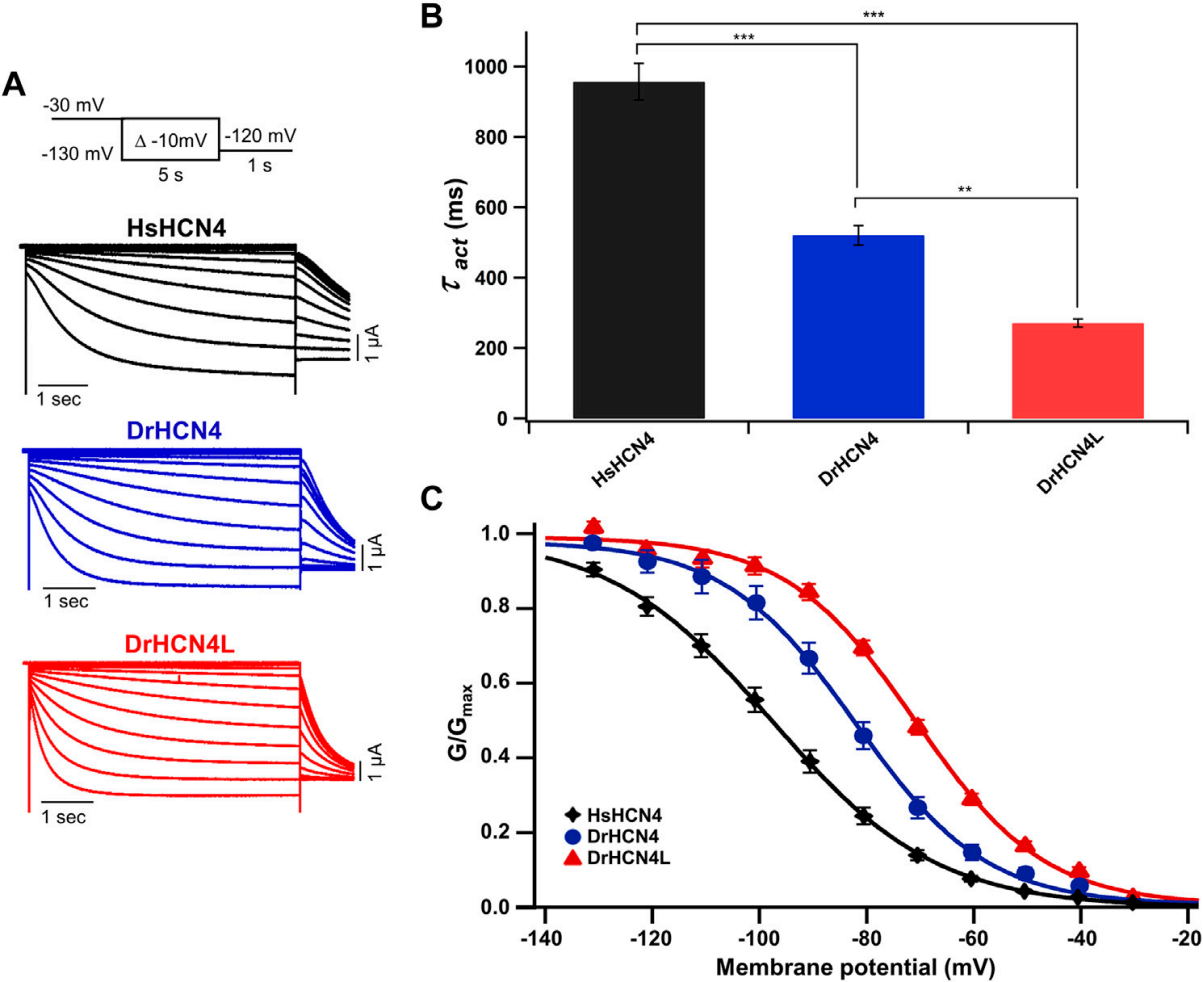
Zebrafish HCN4 channels are hyperpolarization-activated ion channels. **(A)** Representative currents of the human HCN4 channel HsHCN4 (black) and zebrafish HCN4 channels DrHCN4 (blue) and DrHCN4L (red). The holding potential was stepped from −30 mV to −130 mV in 10-mV decrements and held for 5 s at each potential. The inset shows the stimulation protocol. **(B)** Time constants obtained by fitting the activating currents of HsHCN4 (black), DrHCN4 (red) and DrHCN4L (blue) at −130 mV. **(C)** Activation curves (G–V curves) of human and zebrafish HCN4 channels (*n* = 5 for each).

The zebrafish genome contains two HCN4 genes, DrHCN4 on chromosome 18 and DrHCN4L on chromosome 25. Although it is well established that HCN4 channels of other species are hyperpolarization-activated channels, the biophysical properties of the zebrafish HCN4 channels have not been reported. Therefore, we first compared the biophysical properties of zebrafish HCN4 channels (DrHCN4 and DrHCN4L) with those of the human HCN4 channel (HsHCN4). Hyperpolarization-activated inward currents were observed in oocytes expressing DrHCN4 and DrHCN4L, similar to HsHCN4 currents (Figure 1A). The activation time constants (*τ*_act_) of HsHCN4, DrHCN4, and DrHCN4L at −130 mV were 957 ± 52 ms, 521 ± 28 ms, and 272 ± 11 ms, respectively (Figure 1B and Table 1). Therefore, the zebrafish HCN4 channels had faster activation kinetics than the human HCN4 channel, and DrHCN4L showed the fastest activation kinetics. The G-V (conductance-voltage) relationships are shown in Figure 1C. V_1/2_ values of the G-V relationships for HsHCN4, DrHCN4, and DrHCN4L were −97.9 ± 2.1 mV, −83.0 ± 2.4 mV, and −71.1 ± 1.0 mV, respectively (Figure 1C and Table 2). Again, the opening of zebrafish HCN4 channels requires less hyperpolarization than that of the human HCN4, and DrHCN4L showed the most positively-shifted G-V relationship among them.

**TABLE 1:** Activation time constants (τ_*act*_) and *p*-values for the HCN4 channels. N.D.; not determined.

	τ_*act*_ (ms)	P (vs. 4)	P (vs. 4L)
HsHCN4	957 ± 52	< 0.001	< 0.001
DrHCN4	521 ± 27	—	0.003
DrHCN4L	272 ± 11	0.003	—
DrHCN4-4L	357 ± 29	0.070	0.587
DrHCN4L-4	478 ± 63	0.942	0.015
DrHCN4 EA	2117 ± 657	0.072	N.D.
DrHCN4L EA	668 ± 44	N.D.	< 0.001

**TABLE 2:** V_1/2_ (mV) and *p*-values for the HCN4 channels. N.D.; not determined.

	V_1/2_ (mV)	P (vs. 4)	P (vs. 4L)
HsHCN4	−97.9 ± 2.1	<0.001	<0.001
DrHCN4	−83.0 ± 2.4	—	0.002
DrHCN4L	−71.1 ± 1.0	0.002	—
DrHCN4-4L	−77.4 ± 2.0	0.280	0.180
DrHCN4L-4	−86.4 ± 1.7	0.708	<0.001
DrHCN4 EA	−109.1 ± 1.1	<0.001	N.D.
DrHCN4L EA	−94.3 ± 0.8	N.D.	<0.001

### The Cytoplasmic C-Terminal Domain Determines the Voltage Dependence

**FIGURE 2:**
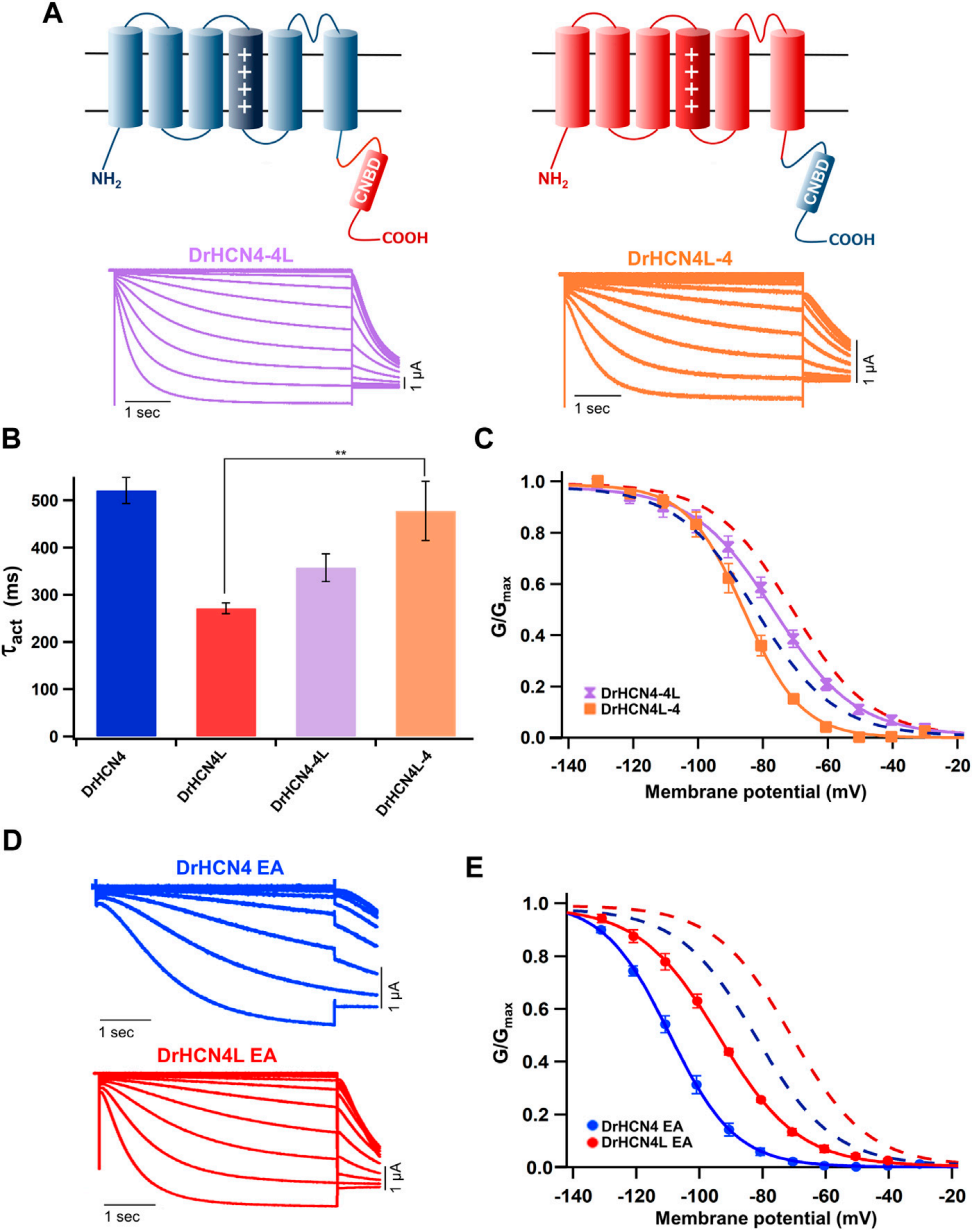
The cytoplasmic C-terminal region of DrHCN4 and DrHCN4L determines the voltage dependence. **(A)** Molecular designs and representative currents of chimeric channels: DrHCN4-4L (left, purple) and DrHCN4L (right, orange). **(B)** Activation time constants (*τ*_act_) at −130 mV of DrHCN4 (blue), DrHCN4L (red), DrHCN4-4L (purple) and DrHCN4L-4 (orange). The currents were fitted with a single exponential. **(C)** Activation curves of the zebrafish chimeric HCN4 channels (*n* = 5). The Boltzmann function was used to fit the curves. Blue and red dashed curves are the activation curves of DrHCN4 and DrHCN4L from Figure 1C. **(D)** Representative current traces of the EA mutants, DrHCN4 EA and DrHCN4L EA. **(E)** Activation curves of the EA mutant channels (*n* = 5). The Boltzmann function was used to fit the curves. Blue and red dashed curves are the activation curves of DrHCN4 and DrHCN4L from Figure 1C.

The voltage sensor movement confers the voltage dependence of HCN channels like other voltage-gated ion channels (Männikkö et al., 2002; Dai et al., 2019). In addition, all HCN channels contain a cyclic nucleotide-binding domain (CNBD) in the intracellular C-terminal region where cyclic nucleotides, primarily cAMP in physiological condition, binds and thereby shifts the G-V relationship of HCN channels towards a depolarized potential. This mechanism is how the activation of G_s_-coupled β1-adrenergic receptors potentiates HCN channels. To determine which part of the zebrafish HCN4 channels is responsible for the different voltage dependence, we made two chimera channels (DrHCN4-4L and DrHCN4L-4) by exchanging the cytoplasmic C-terminal regions of DrHCN4 and DrHCN4L. The molecular design and ionic currents of the chimera channels under a two-electrode voltage clamp are shown in Figure 2A and Supplementary Figure S1 (a red arrow). Activation kinetics of DrHCN4-4L, which has an N-terminal region and a transmembrane region of DrHCN4 with the C-terminal region of DrHCN4L, was 357 ± 29 ms and comparable to that of DrHCN4L (*p* = 0.587), rather than that of DrHCN4 (*p* = 0.070). On the other hand, the activation kinetics of DrHCN4L-4 was 478 ± 63 ms and comparable to that of DrHCN4 (*p* = 0.942), rather than that of DrHCN4L (*p* = 0.015) (Figure 2B and Table 1). The C-terminal exchange of DrHCN4 and DrHCN4L also resulted in a shift of the G-V relationships; V_1/2_ of DrHCN4L-4 chimera was −86.4 ± 1.7 mV and negatively shifted from V_1/2_ of DrHCN4L (−71.1 ± 1.0 mV; *p* < 0.001). V_1/2_ of DrHCN4-4L (−77.4 ± 2.0 mV) was not significantly shifted from that of DrHCN4 (−83.0 ± 2.4 mV; *p* = 0.280), though. (Figure 2C and Table 2). These results suggest that the C-terminal region determines the voltage dependence.

Because the C-terminal region contains the CNBD domain, where cAMP (or other cyclic nucleotides) binds and modulates the gating properties, we hypothesized that the difference in the voltage dependence could be due to a difference in cAMP sensitivity. As it is known that *Xenopus* oocytes contain a high concentration of cAMP, a possible cAMP sensitivity difference could have a high impact on the gating of zebrafish HCN4 channels. To examine if the cAMP sensitivities are different between DrHCN4 and DrHCN4L, we mutated two critical amino acid residues for cAMP binding (R630 and T631 in DrHCN4; R628 and T629 in DrHCN4L) to glutamate and alanine (DrHCN4 EA and DrHCN4L EA). This mutant is known to lose a sensitivity to cAMP (Magee et al., 2015; Fenske et al., 2020). By assuming DrHCN4 EA and DrHCN4L EA lost cAMP-dependent regulation, we expressed them in *Xenopus* oocytes and examined their voltage-dependence and the gating behavior. The EA mutants showed slower activation kinetics and negatively shifted G-V relationships (Figures 2D,E). The G-V shifts were by more than 20 mV, from −83.0 ± 2.4 mV to −109.1 ± 1.1 mV in DrHCN4 EA (*p* < 0.001), and from −71.1 ± 1.0 mV to −94.3 ± 0.8 mV in DrHCN4L EA (*p* < 0.001) (Figure 2E and Table 2). These results suggest that both DrHCN4 and DrHCN4L are markedly sensitive to cAMP.

In conclusion, the gating properties of DrHCN4 and DrHCN4L are primarily dependent on the C-terminal region; however, it is not due to a difference in cAMP sensitivity.

### DrHCN4 and DrHCN4L can Form a Heterotetramer

**FIGURE 3:**
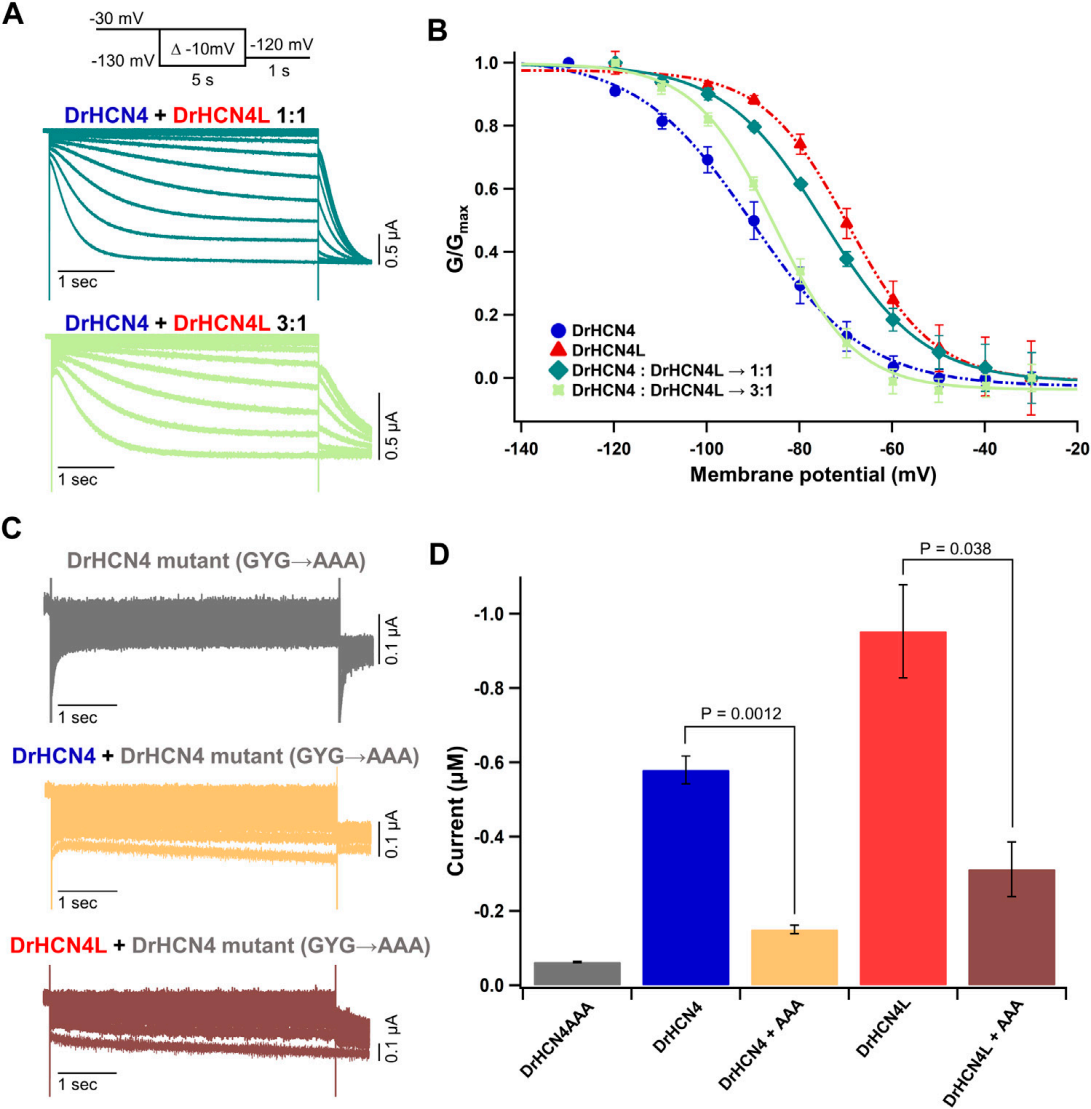
DrHCN4 and DrHCN4L can form a heterotetrametric channel. **(A)** Representative current traces of a mixture of DrHCN4 and DrHCN4L mRNA. The mixed mRNA concentrations were 1:1 (green) and 3:1 (light green). Membrane potential was varied from −30 mV to −130 mV in 10-mV decrements and held for 5 s at each potential. The inset shows the stimulation protocol. **(B)** Activation curves of the mixed channels of DrHCN4 and DrHCN4L. The mixed mRNA concentrations were 1:1 (green) and 3:1 (light green). Blue and red curves are the activation curves of DrHCN4 and DrHCN4L, respectively. The Boltzmann function fits the activation curves (*n* = 5). All data are from the same batch of oocytes. Therefore, the data sets of DrHCN4 and DrHCN4L are different from the datasets used in Figures 1, 2. **(C)** Representative current traces of a dominant-negative mutant with or without wild-type DrHCN4 or DrHCN4L. From top to bottom: DrHCN4 mutant (DrHCN4AAA) only, DrHCN4 (wild-type) + DrHCN4AAA, DrHCN4L (wild-type) + DrHCN4AAA. Wild-type and mutant cRNAs were mixed in a 1:1 ratio with 5 ng each. **(D)** Averaged current amplitudes at −100 mV were plotted as bar graphs [*n* = 5 for each except DrHCN4AAA alone (*n* = 4)].

There are four known isoforms of HCN channels (HCN1-4). A heterotetramer can be formed among different isoforms, and the formation of a heterotetramer diversifies their physiological functions (Chen et al., 2001; Sartiani et al., 2017; Rivolta et al., 2020). For example, the HCN1-HCN2 heteromer is implicated in the pathology of temporal lobe epilepsies (Noam et al., 2011). The HCN1-HCN4 heteromer has been suggested to form native pacemaker channels in the rabbit sinoatrial node (Altomare et al., 2003). Therefore, it is reasonable to speculate that DrHCN4 and DrHCN4L can form a heterotetramer. To verify whether DrHCN4 and DrHCN4L constitute a heterotetramer, a mixture of DrHCN4 and DrHCN4L cRNAs in a ratio of 1:1 (Figure 3A, green) or 3:1 (Figure 3A, light green) was injected into *Xenopus* oocytes. In the G-V relationships, the mixtures of DrHCN4 and DrHCN4L showed intermediate properties between DrHCN4 alone and DrHCN4L alone and could be fitted with a single Boltzmann function (Figure 3B and Table 3).

**TABLE 3:** V_1/2_ (mV) and *p*-values for the homomeric and heteromeric channels.

	V_1/2_ (mV)	P (vs. 4)	P (vs. 4L)
DrHCN4	−88.3 ± 2.6	—	<0.001
DrHCN4L	−69.1 ± 1.6	<0.001	—
DrHCN4:DrHCN4L 1:1	−74.9 ± 1.7	0.002	0.261
DrHCN4:DrHCN4L 3:1	−85.0 ± 1.6	0.625	<0.001

To further demonstrate the presence of the heterotetramer, we made a DrHCN4 mutant “DrHCN4AAA” by replacing glycine-tyrosine-glycine (GYG) amino acid residues located at the selectivity filter with triple alanine (AAA) residues (Supplementary Figure S1, orange box). This mutant is known to function as the dominant-negative mutant in HCN and potassium channels (Xue et al., 2002; Pai et al., 2017; Pitcairn et al., 2017). If more than one DrHCN4AAA mutant subunit(s) is/are included in a DrHCN4L tetramer, it/they will inhibit the ionic current. If that is not the case, wild-type DrHCN4L and DrHCN4AAA mutants independently constitute a channel, and the ionic currents would be observed as if DrHCN4L is expressed alone. First, we confirmed that the DrHCN4AAA mutant alone did not generate ionic currents (Figure 3C, gray). Next, when the DrHCN4AAA mutant was co-expressed with DrHCN4, the current was almost completely suppressed, confirming that the DrHCN4AAA mutant was a dominant-negative mutant (Figure 3C, yellow). Finally, we co-expressed the DrHCN4AAA mutant with DrHCN4L and found that the DrHCN4L current was significantly inhibited (Figures 3C,D). These results further support that DrHCN4 and DrHCN4L can form a heterotetramer.

### Sensitivity of DrHCN4 and DrHCN4L to HCN Inhibitors

**FIGURE 4:**
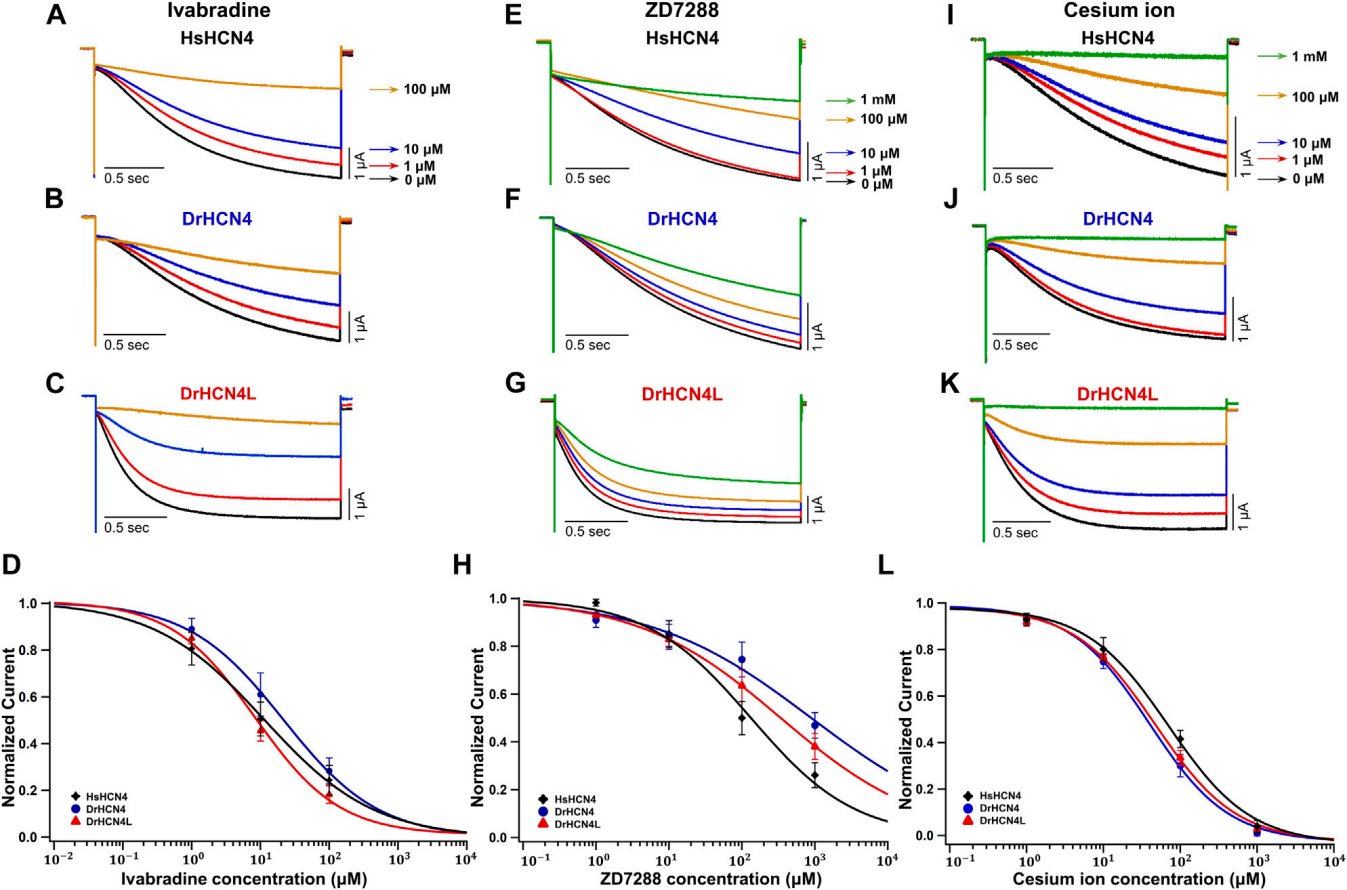
Sensitivities of DrHCN4 and DrHCN4L to HCN inhibitors. **(A–C)** Representative current traces sequentially applied with 0–100 µM of ivabradine. The holding potential was −30 mV and was hyperpolarized to −120 mV for 2 s. **(D)** Ivabradine inhibition curves based on currents of **(A–C)**. DrHCN4 (blue; *n* = 5), DrHCN4L (red; *n* = 4) and HsHCN4 (black; *n* = 4). **(E–G)** Representative current traces sequentially applied with 0 μM–1 mM of ZD7288. The holding potential was −30 mV and was hyperpolarized to −120 mV for 2 s. **(H)** ZD7288 inhibition curves based on currents of **(E–G)**. DrHCN4 (blue; *n* = 5), DrHCN4L (red; *n* = 5) and HsHCN4 (black; *n* = 5). **(I–K)** Representative currents sequentially applied with 0 μM–1 mM of cesium ion. The holding potential was −30 mV and was hyperpolarized to −120 mV for 2 s. **(L)** Cesium ion inhibition curves based on currents of **(I–K)**. DrHCN4 (blue; *n* = 5), DrHCN4L (red; *n* = 5) and HsHCN4 (black; *n* = 5).

We next analyzed the inhibition of DrHCN4 and DrHCN4L by known HCN inhibitors [ivabradine, ZD7288, and cesium ions (Cs^+^)]. Human and zebrafish HCN4 channels were expressed in *Xenopus* oocytes, and the inhibitors were applied to the recording bath. The concentrations of the inhibitors were in the range of 1–100 μM (ivabradine) or 1–1000 μM (ZD7288 and Cs^+^). In the case of ivabradine, the currents of HsHCN4, DrHCN4, and DrHCN4L were similarly inhibited (Figures 4A–C). The IC_50_ values were 38 ± 26 μM for HsHCN4, 31 ± 15 μM for DrHCN4, and 10 ± 3 μM for DrHCN4L (Figure 4D; Table 4). In the case of ZD7288, the IC_50_ values were 167 ± 56 μM for HsHCN4, 699 ± 216 μM for DrHCN4, and 596 ± 382 μM for DrHCN4L (Figures 4E–H; Table 4). In the case of Cs^+^, the IC_50_ values were 69 ± 14 μM for HsHCN4, 44 ± 11 μM for DrHCN4, and 48 ± 7 μM for DrHCN4L (Figures 4I–L; Table 4).

**TABLE 4:** IC_50_ (μM) and *p*-values for the HCN4 inhibitors.

		IC_50_ (μM)	P
Ivabradine	HsHCN4	37.6 ± 25.6	0.958 (Hs4 vs Dr4)
	DrHCN4	30.9 ± 15.3	0.674 (Dr4 vs Dr4L) 0.544 (Hs4 vs Dr4L)
	DrHCN4L	10.4 ± 2.6
ZD7288	HsHCN4	167 ± 56	0.255 (Hs4 vs Dr4)
	DrHCN4	699 ± 216	0.950 (Dr4 vs Dr4L) 0.435 (Hs4 vs Dr4L)
	DrHCN4L	596 ± 383
Cesium ion	HsHCN4	68.8 ± 14.0	0.297 (Hs4 vs Dr4)
	DrHCN4	44.3 ± 11.2	0.964 (Dr4 vs Dr4L) 0.420 (Hs4 vs Dr4L)
	DrHCN4L	48.3 ± 6.9

In conclusion, the HCN inhibitors also inhibit zebrafish HCN4 channels as well.

### HCN Inhibitors Reduce the Heart Rate of Developing Zebrafish Embryos

**FIGURE 5:**
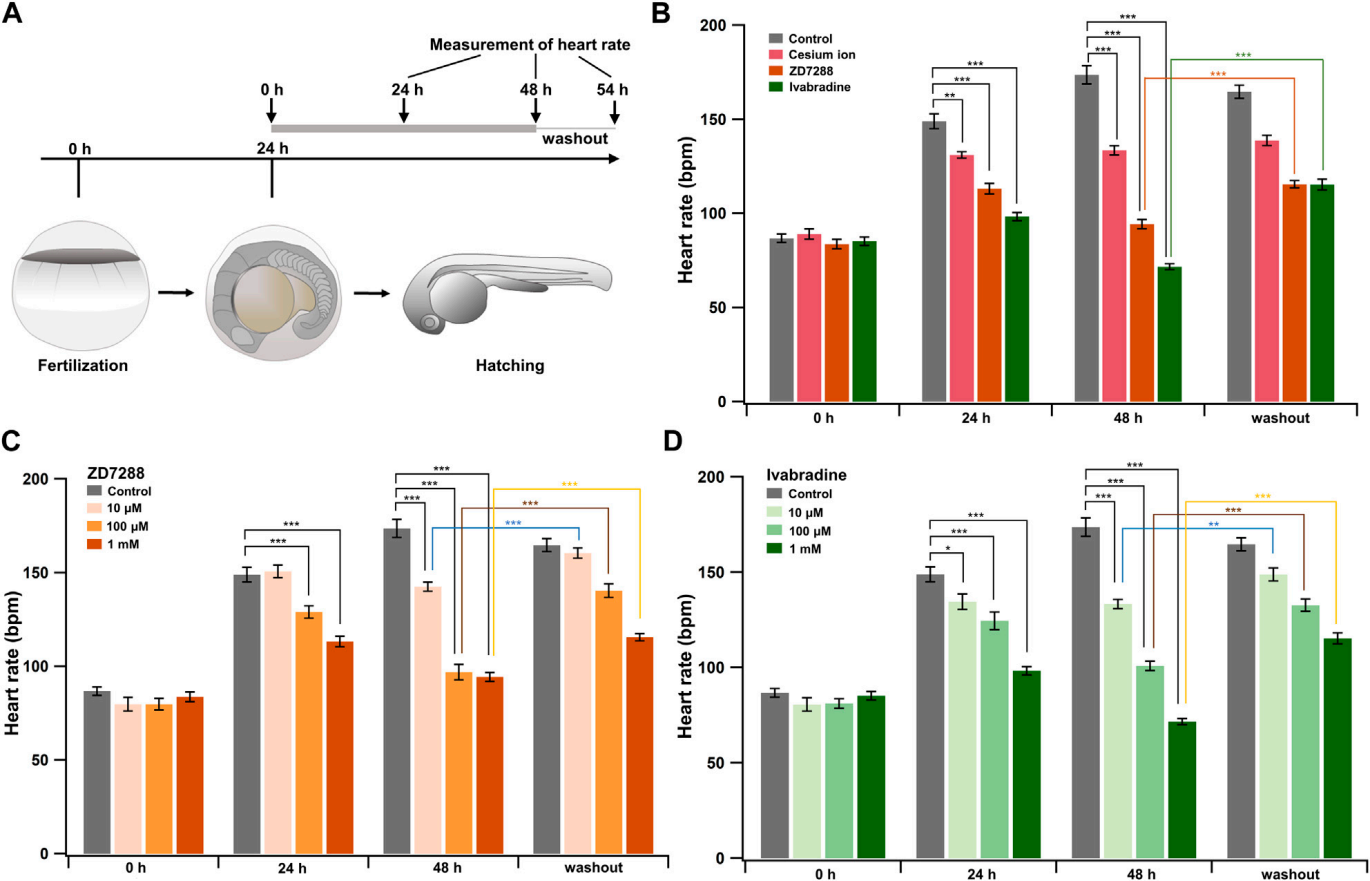
HCN inhibitors reduce heart rate in developing zebrafish embryos. **(A)** Experimental method. Inhibitors were administered in the egg water at 24 hpf, and this time point was designated as “0 h”. After administering the inhibitors, heart rate was measured at 24 h (24 h) and 48 h (48 h). Zebrafish were then transferred back to the inhibitor-free egg water, and their heart rates were measured 6 h later (washout). **(B)** Changes in heart rate with time in zebrafish before (control; grey; *n* = 10) and after treatment with 1 mM cesium ion (pink; *n* = 10), ZD7288 (orange; *n* = 10) and ivabradine (green; *n* = 10) inhibitors. Heart rates before (0 h) and at 24 h (24 h) and 48 h (48 h), after application of the inhibitors and at 6 h after washing out the inhibitors (washout) are shown as bar graphs. Dunnett’s test was used for statistical analysis, and Student’s *t*-test was used to compare before and after “washout.” **(C)** Changes in heart rate with time in zebrafish treated with ZD7288 before (gray; *n* = 10) and after treatment with 10 μM (light orange; *n* = 10), 100 μM (orange; *n* = 10), and 1 mM (dark orange; *n* = 10). Dunnett’s test was used for statistical analysis, and Student’s t-test was used to compare before and after washout. **(D)** Changes in heart rate with time in zebrafish treated with ivabradine before (gray; *n* = 10) and after treatment with 10 μM (thin green; *n* = 10), 100 μM (light green; *n* = 10) and 1 mM (dark green; *n* = 10). Dunnett’s test was used for statistical analysis, and Student’s *t*-test was used to compare before and after washout.

Ivabradine is used to treat chronic heart failure and other disorders as it slows heart rate by inhibiting HCN channel currents in the sinoatrial node (Swedberg et al., 2010). Next, we applied the HCN inhibitors, including ivabradine, to developing zebrafish embryos to see if they were equally efficient in lowering heart rate as they are humans. The HCN inhibitors (Cs^+^, ZD7288, and ivabradine) at a final concentration of 1 mM were applied to the egg water in which fish were kept, starting at 24 h post-fertilization (hpf). Heart rates were recorded before (0 h) and at 24 h (24 h) and 48 h (48 h) after the application of the inhibitors. Zebrafish embryos were then brought back to the inhibitor-free water, and their heart rates were recorded after 6 h (54 h: “washout”) (Figure 5A). In the case of developing zebrafish embryos without an inhibitor (control), heart rate increased from 87 ± 2 bpm [0 h (24 hpf)] to 149 ± 4 bpm [24 h (48 hpf)] and 174 ± 5 bpm [48 h (72 hpf)] during the span (Figure 5B, gray bars). All HCN inhibitors significantly reduced the heart rate of zebrafish at 24 and 48 h after drug application (Figure 5B). After the subsequent washout of HCN inhibitors, the heart rates in both the ZD7288 and ivabradine groups (but not the Cs^+^ group) recovered (Figure 5B and Table 5). We then examined the concentration dependence of the effects of ivabradine and ZD7288 (Figures 5C,D). ZD7288 significantly lowered heart rate, except for a concentration of 10 μM at 24 h (Figure 5C). Ivabradine significantly suppressed heart rate at all concentrations and time points, and the effect was more potent at higher concentrations and longer time (Figure 5D). Furthermore, the heart rates in all ZD7288- and ivabradine-treated zebrafish were significantly recovered by washing out the inhibitors (Figures 5C,D and Table 5).

**TABLE 5:** Inhibitory effects on heart rates (bpm) and *p*-values in developing zebrafish embryos.

		Heart rate (bpm)	P (vs. control)
0 h (24 hpf)	Control	87 ± 2	—
	Cesium ion (1 mM)	89 ± 1	0.999
	ZD7288 (10 μM)	81 ± 4	0.574
	ZD7288 (100 μM)	80 ± 3	0.357
	ZD7288 (1 mM)	84 ± 3	0.960
	Ivabradine (10 μM)	81 ± 3	0.499
	Ivabradine (100 μM)	81 ± 2	0.589
	Ivabradine (1 mM)	85 ± 2	0.999
24 h	Control	149 ± 4	—
	Cesium ion (1 mM)	131 ± 2	0.002
	ZD7288 (10 μM)	150 ± 3	0.999
	ZD7288 (100 μM)	129 ± 1	<0.001
	ZD7288 (1 mM)	113 ± 3	<0.001
	Ivabradine (10 μM)	134 ± 4	0.021
	Ivabradine (100 μM)	124 ± 5	<0.001
	Ivabradine (1 mM)	98 ± 2	<0.001
48 h	Control	174 ± 5	—
	Cesium ion	133 ± 2	<0.001
	ZD7288 (10 μM)	142 ± 2	<0.001
	ZD7288 (100 μM)	97 ± 4	<0.001
	ZD7288 (1 mM)	94 ± 2	<0.001
	Ivabradine (10 μM)	133 ± 2	< 0.001
	Ivabradine (100 μM)	101 ± 2	<0.001
	Ivabradine (1 mM)	72 ± 2	<0.001
		**Heart rate (bpm)**	**P (vs. 48 h)**
Washout	Control	165 ± 3	—
	Cesium ion	139 ± 3	0.168
	ZD7288 (10 μM)	160 ± 3	<0.001
	ZD7288 (100 μM)	140 ± 4	<0.001
	ZD7288 (1 mM)	115 ± 2	<0.001
	Ivabradine (10 μM)	149 ± 3	0.002
	Ivabradine (100 μM)	133 ± 3	<0.001
	Ivabradine (1 mM)	115 ± 3	<0.001

These results suggest that ivabradine and ZD7288 reduce heart rate in developing zebrafish embryos. The inhibitory effect of cesium ions on zebrafish heart rate was less than that expected from the results for *Xenopus* oocytes.

### HCN4 Channel Knockdown Transiently Reduces Heart Rate and Induces Pericardial Edema in Zebrafish

**FIGURE 6:**
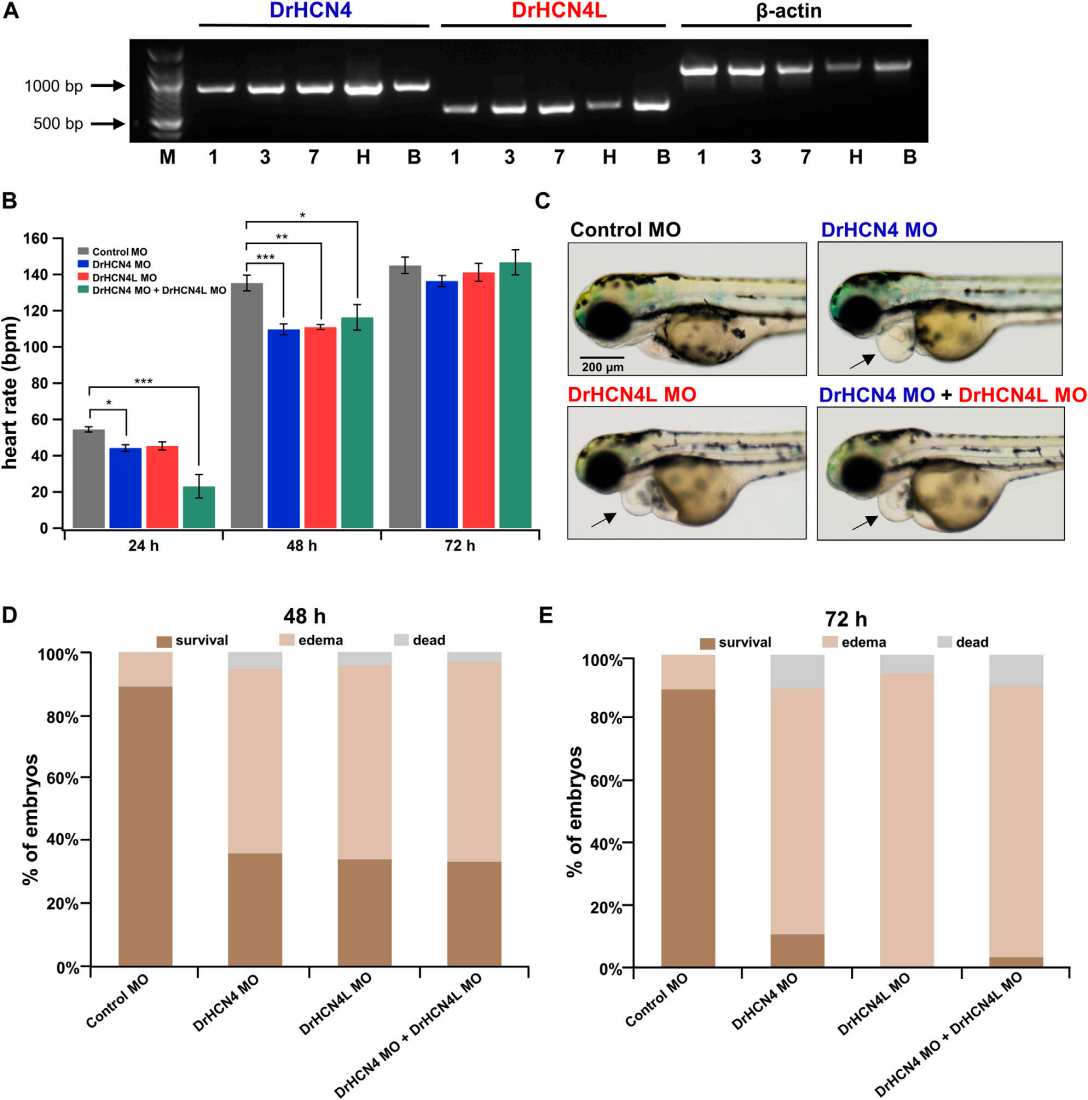
Effects of HCN4 channel knockdown on zebrafish. **(A)** Expression of DrHCN4 and DrHCN4L in one (1), three (3), and seven (7) day embryos/larvae as well as in the heart (H) and brain (B) of adult fish was confirmed by RT-PCR. Arrows indicate the DNA ladder markers of 1,000 bp and 500 bp. **(B)** Heart rates at 24, 48, and 72 h are shown in bar graphs for 200 μM MO-injected zebrafish embryos: control MO (*n* = 20), DrHCN4 MO (*n* = 10), DrHCN4L MO (*n* = 10), and mixed DrHCN4 and DrHCN4L MO (*n* = 10). Statistical analysis was performed by Dunnett’s test. **(C)** Representative examples of zebrafish injected with control MO, DrHCN4 MO, DrHCN4L MO, and mixed DrHCN4 and DrHCN4L MO. Zebrafish with DrHCN4 MO, DrHCN4L MO, and mixed DrHCN4 and DrHCN4L MO showed pericardial edema (black arrows). Bar, 200 μm. **(D)** Ratios of normal embryos, pericardial edema, and death at 48 h after injection of 200 µM MO: control MO (*n* = 136), DrHCN4 MO (*n* = 75), DrHCN4L MO (*n* = 34), and mixed DrHCN4 and DrHCN4L MO (*n* = 30). **(E)** Pericardial edema and death ratios at 72 h after injection of 200 µM MO. Control MO (*n* = 136), DrHCN4 MO (*n* = 75), DrHCN4L MO (*n* = 34), and mixed DrHCN4 and DrHCN4L MO (*n* = 30).

Since DrHCN4 and DrHCN4L have similar sensitivities to the HCN inhibitors, individual gene knockdowns would be required for evaluating each channel’s role in heart rate regulation. Before knocking down each gene, we first confirmed that both HCN4 channels (DrHCN4 and DrHCN4L) were expressed in day 1, day 3, and day 7 embryos and in the heart and brain of adult fish (Figure 6A). We next investigated whether the knockdown of each gene affects heart rate in zebrafish. The expression of each gene was knocked down by using antisense morpholino (MO), which is commonly used in zebrafish (Blum et al., 2015; Jou et al., 2017; Fujii et al., 2020). MO solutions were injected into 1-cell embryos of zebrafish. First, we confirmed that water or control MO injection did not affect heart rate (Supplementary Figure S2). Embryos were injected with control MO, DrHCN4 MO, and DrHCN4L MO at concentrations of 200 μM or with a mixture of DrHCN4 MO and DrHCN4L MO (200 μM for each). At 24 h after injection of MO, there was a slight decrease of heart rate in embryos injected with DrHCN4 MO (44 ± 2 bpm, *p* = 0.049) and DrHCN4L MO (45 ± 2 bpm, *p* = 0.092) compared to the heart rate in embryos injected with control MO (54 ± 1 bpm). The heart rate of zebrafish injected with a mixture of DrHCN4 MO and DrHCN4L MO was significantly reduced to 23 ± 6 bpm (*p* < 0.001; Figure 6B and Table 6). After 48 h, heart rate was considerably lower in all three groups compared to that in the control group (135 ± 4 bpm): DrHCN4 MO (110 ± 3 bpm, *p* < 0.001), DrHCN4L MO (111 ± 1 bpm, *p* = 0.001), and mixture of DrHCN4 MO and DrHCN4L MO (116 ± 7 bpm, *p* = 0.014). However, at 72 h after MO injection, heart rate in all three groups had returned to the same level as that in the control group (145 ± 5 bpm): DrHCN4 MO (136 ± 3 bpm, *p* = 0.509), DrHCN4L MO (141 ± 5 bpm, *p* = 0.921), and mixture of DrHCN4 MO and DrHCN4L MO (147 ± 7 bpm, *p* = 0.991) (Figure 6B and Table 6).

**TABLE 6:** Inhibitory effects of antisense MO on heart rates (bpm) and *p*-values in developing zebrafish embryos.

(h)		Heart rate (bpm)	P (vs. control)
24	Control MO	54 ± 1	—
	DrHCN4 MO	44 ± 2	0.049
	DrHCN4L MO	45 ± 2	0.092
	DrHCN4 MO + DrHCN4L MO	23 ± 6	< 0.001
48	Control MO	135 ± 4	—
	DrHCN4 MO	110 ± 3	< 0.001
	DrHCN4L MO	111 ± 1	0.001
	DrHCN4 MO + DrHCN4L MO	116 ± 7	0.014
72	Control MO	145 ± 5	—
	DrHCN4 MO	136 ± 3	0.509
	DrHCN4L MO	141 ± 5	0.921
	DrHCN4 MO + DrHCN4L MO	147 ± 7	0.991

We noticed that zebrafish embryos injected with HCN4 MO tended to exhibit pericardial edema (Figure 6C). Pericardial edema is often observed in embryonic zebrafish with heart failure (Narumanchi et al., 2021). We determined the incidence of edema in zebrafish injected with 200 μM MO. We only examined zebrafish embryos that survived for the first 24 h after MO injection, and the rates of edema occurrence were determined at 48 and 72 h after injection. At 48 h after injection, 59, 62, and 63% of the embryos injected with DrHCN4 MO, DrHCN4L MO, and a mixture of DrHCN4 MO and DrHCN4L MO, respectively, showed pericardial edema (Figure 6D). After 72 h, the proportions of embryos with edema increased to 79, 94, and 87% (Figure 6E). These results suggested that both DrHCN4 and DrHCN4L are required for proper cardiac function at early stages.

## Discussion

### Biophysical Properties of DrHCN4 and DrHCN4L Channels

In this study, we first analyzed the biophysical properties of DrHCN4 and DrHCN4L and compared them to the biophysical properties of HsHCN4. DrHCN4L showed the fastest activation kinetics, DrHCN4 showed the second-fastest activation kinetics, and HsHCN4 showed the slowest activation kinetics (Figure 1B). The G-V curves showed similar tendencies; DrHCN4L showed the most positively shifted G-V curve (Figure 1C). The chimera experiments suggest that the cytoplasmic region might determine their voltage dependence (Figure 2). It is known that the intracellular cyclic nucleotides cAMP activates HCN channels (Zagotta et al., 2003; Alvarez-Baron et al., 2018; Saponaro et al., 2021). According to the EA mutant experiments, however, the difference in the voltage dependence is not due to different cAMP sensitivities (Figures 2D,E). Although we did not further explore the causes of different gating properties here, we noticed some unique amino acid residues only in DrHCN4L around the CNBD domain (Supplementary Figure S1). Those unique amino acid residues might change the gating properties and accelerate the activation kinetics and positively shift the voltage dependence in DrHCN4L.

Mammalian HCN1, HCN2, and HCN4 are known to form a heterotetramer (Chen et al., 2001; Rivolta et al., 2020). Therefore, it is highly likely that DrHCN4 and DrHCN4L form a heterotetramer. By generating a dominant-negative mutant (DrHCN4AAA) (Figure 3C), we confirmed that the zebrafish HCN4 channels (DrHCN4 and DrHCN4L) are also capable of forming a heterotetramer, at least in *Xenopus* oocytes. Using a similar HCN4 dominant-negative mutant, it has been shown that HCN4 channels play an important role in the early development of *Xenopus laevis* (Pai et al., 2017; Pitcairn et al., 2017). This dominant-negative mutant of DrHCN4 can be used as a tool to elucidate the early developmental and physiological roles of DrHCN4 and DrHCN4L in zebrafish, as in the case of *Xenopus laevis*.

### Pharmacological Properties of Zebrafish HCN4 Channels

We compared the sensitivities of HCN4 channels (HsHCN4, DrHCN4, and DrHCN4L) to common HCN channel inhibitors (ZD7288, ivabradine, and Cs^+^) (Figure 4). Cs^+^, ZD7288, and ivabradine similarly inhibited zebrafish HCN4 channels and human HCN4 channels expressed in *Xenopus* oocytes (Figure 4 and Table 4).

Using developing zebrafish embryos, we investigated whether the administration of HCN inhibitors (ZD7288, ivabradine, and Cs^+^) in the egg water affected heart rate (Figure 5). The results showed that HCN inhibitors, especially ivabradine, effectively reduced the heart rate of zebrafish. However, the effects of these blockers were not immediately apparent, and a significant difference was seen only after several hours. In some previous studies using other compounds, there was also a waiting period of 4–24 h before cardiotoxicity was observed, partly because compounds must be taken up through the epidermis without mouth opening at the early stages (Bauer et al., 2021). In recent pharmacological experiments using adult zebrafish, intraperitoneal injection of zatebradine reduces maximum heart rate by 38–65% depending on the test temperatures (Marchant and Farrell, 2019). Isolated adult zebrafish heart is also effectively blocked by bath-applications of ivabradine and Cs^+^ to by 86 and 73%, respectively (Stoyek et al., 2022). Therefore, uptake (or excretion) might be the reason for ineffectiveness of Cs^+^ here.

We have to mention that the HCN inhibitors may affect other cardiac ion channels such as voltage-gated sodium channels and hERG, especially at high concentration (Wu et al., 2012; Haechl et al., 2019; Hackl et al., 2022). Inhibiting these “off-target” ion channels by the HCN inhibitors could affect heart rate and might explain why ivabradine and ZD7288 had larger effects than cesium or than the morpholino experiments.

### Physiological Functions of DrHCN4 and DrHCN4L in the Heart

To discriminate possible physiological functions and roles of DrHCN4 and DrHCN4L in the heart, we tried to suppress the expression of each gene. In the present study, we used antisense morpholino (MO) to knock down DrHCN4 and DrHCN4L genes because MO knockdown is commonly used in zebrafish (Blum et al., 2015; Fujii et al., 2020; Minhas et al., 2021).

Injection of MO into zebrafish 1-cell embryos resulted in a transient reduction in heart rate (Figure 6). In this knockdown experiment performed using MO, heart rate was temporarily reduced but returned to almost the same level as that in the wild type at 72 hpf. One possible explanation of this transient nature of heart rate reduction is due to compensation mechanism by other HCN channel genes or other pacemaker-related ion channel genes such as T-type Ca^2+^ channels. Another possibility is degradation of MO. Unfortunately, we could not exclude the latter possibility due to lack of detection tools like antibodies. Therefore, we discuss the following section by assuming MO is effective throughout the experimental time window (∼72 hpf).

Jou et al. previously examined the MO against zebrafish HCN4 and observed a similar heart rate reduction at 36–40 hpf (Jou et al., 2017). However, they did not examine the MO-injected embryos at later stages; therefore, they do not describe the heart rate recovery we observed at 72 h. In addition, the MO used in their study was only for DrHCN4, while ours is the first study in which heart rate was assessed by using an MO targeting both DrHCN4 and DrHCN4L. At 48 hpf, the heart rate was similarly reduced regardless of the knockdown gene (Figure 6B). However, other HCN channels, such as HCN1 and HCN2, may be expressed in zebrafish hearts (Ludwig et al., 1999). In addition, the “Ca^2+^ clocks” mechanism also contributes to the automaticity of the zebrafish heart (Stoyek et al., 2022). Therefore, heart rate may not be determined only by HCN4 and HCN4L expression. von der Heyde et al. used CRISPR/Cas9 targeting DrHCN4 and DrHCN4L to examine the effects of mutations on heart rate and heart rate variability in zebrafish (von der Heyde et al., 2020). They “unexpectedly” observed a higher heart rate at 2 days post-fertilization (dpf) and 5 dpf in zebrafish with nonsense mutations in both DrHCN4 alleles and speculated that the higher heart rate was facilitated by compensatory expression of HCN channels (von der Heyde et al., 2020). Although we do not know why they did not observe heart rate reduction with DrHCN4 knockout by CRISPR/Cas9, the heart rate recovery we observed may be due to a similar compensation mechanism by HCN expressions, or other pacemaker mechanisms including T-type Ca^2+^ channels or calcium clock-related proteins.

We also found that knockdown of the HCN4 genes by MO tended to induce pericardial edema (Figures 6D,E). Therefore, DrHCN4 and DrHCN4L may play an important role in controlling heart rate but also in normal function of the heart in the early stages. On the other hand, no pericardial edema was observed in zebrafish treated with ivabradine or ZD7288. Unlike the MO knockdown experiment, the HCN4 channel proteins are present in the plasma membrane during inhibition. Therefore, the presence of the HCN4 channel protein, not the ionic currents, might be required in normal function of the heart at the early stages.

One of the limitations of this study is that our findings may only be applied to embryonic zebrafish. Because zebrafish are transparent during the early stages, it is a great advantage to use them for exploring the physiological functions of organs like the heart *in vivo*. However, the physiological functions we studied here could differ in adult fish. At least, it has been reported that HCN4 proteins are expressed in putative pacemaker cells in adult zebrafish (Stoyek et al., 2015). We ourselves confirmed that both DrHCN4 and DrHCN4L were expressed in the adult heart and brain (Figure 6A). Still, future works would be required to verify that DrHCN4 and/or DrHCN4L are also physiologically important in adult fish.

Another possible limitation of this study is temperature. As we performed all experiments at room temperature, including two-electrode voltage-clamp and heart rate measurement. However, it is known that temperature has a substantial effect on heart rate and action potentials in zebrafish (Lin et al., 2014; Rayani et al., 2018). It is also known that HCN channels are somewhat temperature-dependent: the value of Q_10_ (folds of increase for every 10°C) of activation and deactivation kinetics is 3–5, and the Q_10_ of current amplitude is approximately 2 (Magee, 1998; Elinder et al., 2006). Our experimental conditions (approximately 25°C) are about 3°C lower than the breeding condition (28°C), which could slow activation kinetics up to 1.6 -fold and reduce amplitude to about 80%. Therefore, temperature differences must be considered if the present study’s data is to be translated into *in vivo* study.

In conclusion, the results of our study suggest that DrHCN4 and DrHCN4L play functional roles in heart rate regulation and possibly normal cardiac function during early stages. Therefore, when using zebrafish to create transgenic lines or disease models of HCN4 channels, it should be taken into consideration that two HCN4 channels are functional in zebrafish.

## Data Availability

The original contributions presented in the study are included in the article/Supplementary Material, further inquiries can be directed to the corresponding author.

## Supplementary Material

Supplementary Figure S1 Sequence alignment of human and zebrafish HCN4 channels. The amino acid sequences were aligned using Clustal Omega (Sievers et al., 2011), and are shown using ESPript3 (Robert and Gouet, 2014). The S1 to S6 segments and CNBD domain from human HCN4 structure (PDB: 6GYO) are labeled above the alignments. The selective filter “GYG motif” is highlighted with an orange square. The residues involved in the cAMP binding and mutated in this study are highlighted with red dots. The red arrow indicates where the chimera constructs (4-4L and 4L-4 in Figure 2) are switched.

Supplementary Figure S2 Control MO or water injection do not affect heart rate. Averaged heart rates of uninjected, water-injected, and control MO-injected zebrafish embryos (52 hpf).
