# Supplementary figures and images for: Two HCN4 Channels Play Functional Roles in the Zebrafish Heart
Source: Front Physiol. 2022 Jun 30;13:901571. doi: 10.3389/fphys.2022.901571 (PMC9281569; doi:10.3389/fphys.2022.901571)

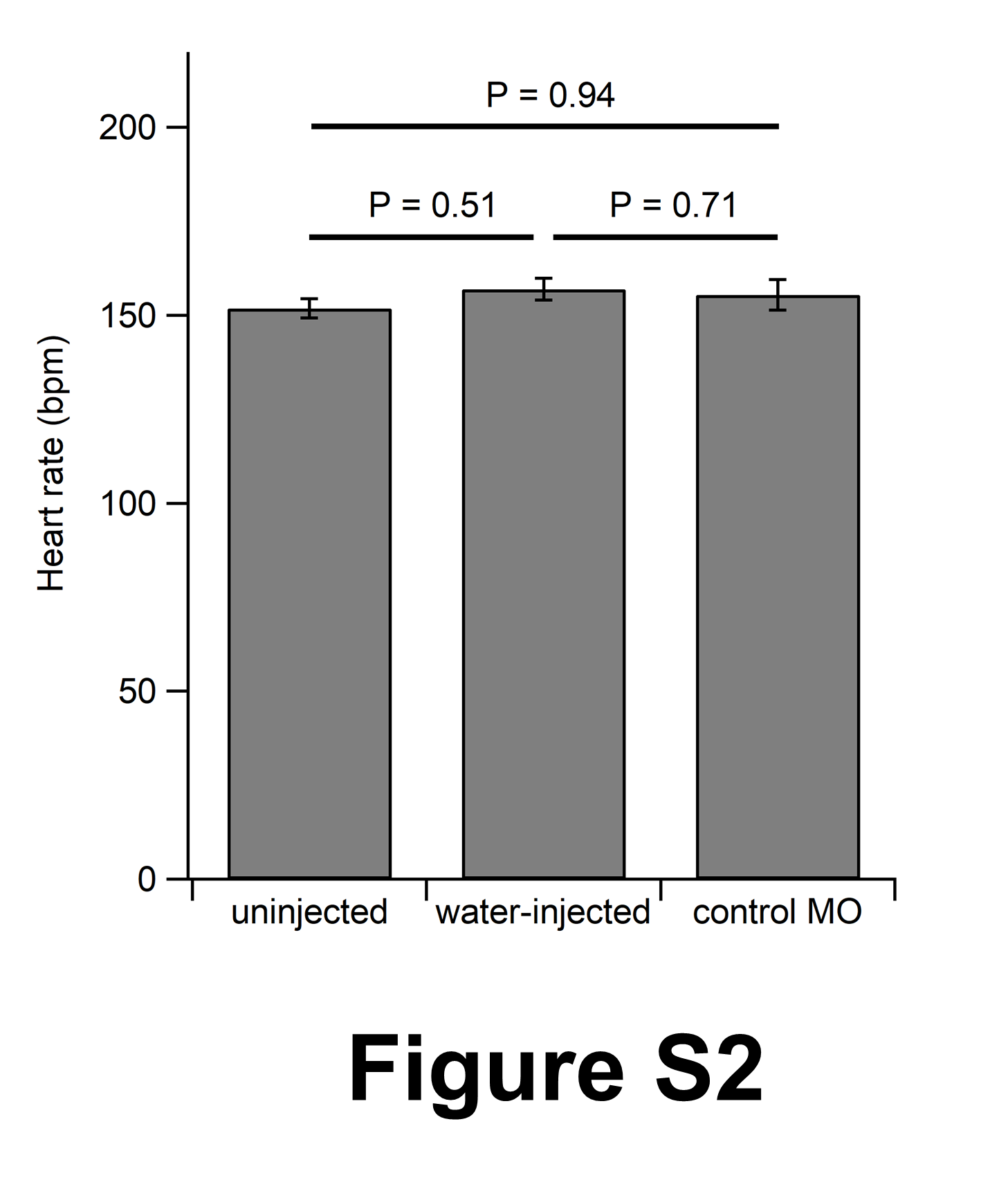

Supplement: Supplementary file 1 [file Image2.TIF]
