# Supplementary material for: Two HCN4 Channels Play Functional Roles in the Zebrafish Heart
Source: Front Physiol. 2022 Jun 30;13:901571. doi: 10.3389/fphys.2022.901571 (PMC9281569; doi:10.3389/fphys.2022.901571)
Supplement: Supplementary file 2 [file Image1.PDF]

```
         1        10        20        30        40        50        60        70        80
HsHCN4   MDKLPPSMRKRLYSLPQQVGAKAWIMDEEEDAEEEGAGGRQDPSRRSIRLRPLPSPSPSAAAGGTESRSSALGAADSEGP
DrHCN4   MDRLHSSMRKRLYSLPQHIGQKASIMGDAEDSD.......KDTRRKSIRMKPLPTSGEIK......SGESAIMETDI...
DrHCN4L  MHRVHSSMRKRLYSLPHQFGHKGAMTRDGEDSD.......KDTRRKSVKMKHFPSPSSTGSSKGVCEAKSGDAETDV...

                  90       100       110       120       130       140       150       160
HsHCN4   ARGAGKSSTNGDCRRFRGSLASLGSRGGGSGGTGSGSSHGHLHDSAEERRLIAEGDASPGEDRTPPGLAAEPERPGASAQ
DrHCN4   .GRPVKTSSNGDCRRFRGSLSSITSRHVH............DSDVAEERRLITDGEVTPSEESPP.GAAGGGAEPGAQSG
DrHCN4L  .GRPFRTNVNGDCRRFRGSLSSLTSRHAAP...........DADLAEQRRLIGD..ASPEDDGPS.EQPGPGESPGAKGG

                  170       180       190       200       210       220       230
HsHCN4   PAASP.....PPPQQPPQPASASCEQPSVDTAIKVEGGAAAGDQILPEAEVRLGQAGFMQRQFGAMLQPGVNKFSLRMFG
DrHCN4   GGGGEEAAGSSQHQGIPD.........QQAGFIKLEG....IEQILPD.DERYYQAGFMHRQFGAMLQPGVNKFSLRMFG
DrHCN4L  RGDGAQ.......QAPSE.........EQSTFVKLEG....IDQILPD.DERMYQAGFMHRQFGAMLQPGVNKFSLRMFG

                                       S1                          S2
            240       250       260       270       280       290       300       310
HsHCN4   SQKAVEREQERVKSAGFWIIHPYSDFRFYWDLTMLLLMVGNLIIIPVGITFFKDENTTPWIVFNVVSDTFFLIDLVLNFR
DrHCN4   SEKAVEREQERVKSAGFWIIHPYSDFRFYWDLTMLLLMVGNLIIIPVGITFFKDEHTPPWIVFNVVSDTFFLMDLVLNFR
DrHCN4L  SEKAVEHEQERVKSAGFWIIHPYSDFRFYWDLIMLLLMVGNLIIIPVGITFFKDEHTPAWIVFNVVSDTFFLVDLVLNFR

                                 S3                                   S4
            320       330       340       350       360       370       380       390
HsHCN4   TGIVVEDNTEIILDPQRIKMKYLKSWFMVDFISSIPVDYIFLIVETRIDSEVYKTARALRIVRFTKILSLLRLLRLSRLI
DrHCN4   TGIVKEDNAEIILDPQQIKIKYLRSWFVVDFISSIPVDYIFLIVETRIDSDFYKTARALRIVRFTKILSLLRLLRLSRLI
DrHCN4L  TGIVKEDSTEIILDPQQIKVRYLRSWFAVDFLSSIPVDYIFLIVETRIDSDFYKTARALRIVRFTKILSLLRLLRLSRLI

                                  S5
            400       410       420       430       440       450       460       470
HsHCN4   RYIHQWEEIFHMTYDLASAVVRIVNLIGMMLLLCHWDGCLQFLVPMLQDFPDDCWVSINNMVNNSWGKQYSYALFKAMSH
DrHCN4   RYIHQWEEIFHMTYDLASAMVRIVNLIGMMLLLCHWDGCLQFLVPMLQDFPADCWVAKNKMVNDTWGQQYSYALFKAMSH
DrHCN4L  RYIHQWEEIFHMTYDLASAMVRIVNLIGMMLLLCHWDGCLQFLVPMLQDFPSDCWVAKNKMVNDTWGQQYSYALFKAMSH

                              S6
            480       490       500       510       520       530       540       550
HsHCN4   MLCIGYGRQAPVGMSDVWLTMLSMIVGATCYAMFIGHATALIQSLDSSRRQYQEKYKQVEQYMSFHKLPPDTRQRIHDYY
DrHCN4   MLCIGYGMYPPVGMTDVWLTILSMIVGATCYAMFVGHATALIQSLDSSRRQYQEKYKQVEQYMSFHKLPADMRQRIHDYY
DrHCN4L  MLCIGYGMYPPVGMTDVWLTILSMIVGATCYAMFVGHATALIQSLDSSRRQYQEKYKQVEQYMSFHKLPADMRQRIHDYY

            560       570       580       590       600       610       620       630
HsHCN4   EHRYQGKMFDEESILGELSEPLREEIINFNCRKLVASMPLFANADPNFVTSMLTKLRFEVFQPGDYIIREGTIGKKMYFI
DrHCN4   EHRYQGKMFDEESILGELNEPLREEIINFNCRKLVASMPLFANADPNFVTSMLTKLRFEVFQPGDYIIREGTIGKKMYFI
DrHCN4L  EHRYQGKMFDEESILGELNEPLREEIISFNCRKLVATMPLFANADPNFVTSMLTKLRFEVFQPGDYIIREGTVGKKMYFI

                                              CNBD
            640       650       660       670       680       690       700       710
HsHCN4   QHGVVSVLTKGNKETKLADGSYFGEICLLTRGRRTASVRADTYCRLYSLSVDNFNEVLEEYPMMRRAFETVALDRLDRIG
DrHCN4   QHGVVSVLTKGNKETKLSDGSYFGEICLLTRGRRTASVRADTYCRLYSLSVDNFNEVLEEYPMMRRAFETVALDRLDRIG
DrHCN4L  QHGVLTVLTKGSKETKISDGSYFGEICLLTRGRRTASVRADTYCRLYSLSVDHFNEVLEEYPMMRRAFETVALDRLDRIG
```

```
           720       730       740       750       760       770       780
HsHCN4   KKNSILLHKVQHDLNSGVFNYQENEIIQQIVQHDREMAHCAHRVQAAASA......TPTPTPVIWTPLIQAPLQAAAATT
DrHCN4   KKNSILQHKVQHDLNSGVLNYQESEIIQQIVQHDRDMAHCANLLQSPSPPAPPS.....PTPVIWAPLIQAPLQAAAATT
DrHCN4L  KRNSVLQHKVQRDLNSGVLNYQENEIIQQIVQHDRDMAHCAHLMQTPPPPPHPAPTPSSHTPVIWAPLIQAPLQAAAATT

         790       800       810       820       830       840       850       860
HsHCN4   SVAIALTHHPRLPAAIFRPPPGSGLGNLGAGQTPRHLKRLQSLIPSALGSA.SPASSP....SQVDTPSSSSFHIQQLAG
DrHCN4   SVAIALTHHPHLPATLFRPPVSL.LGSR..NEPPSRLKRFQTVAPRTGSTTDSPSTSPSKLHSGVDTPLLASLHTQHSSV
DrHCN4L  SVAIALTRHPHLPHTLFRPPVPR.LGSL..KDQPGYFKRFPTALSGADTFG.GSSSTSSQFSSCIETPILDTLRAERSST

              870       880       890       900
HsHCN4   FSAPAGLSPLLPSSSSSPPPGACGSPSAPTPSAGVAATTIAG......................................
DrHCN4   ...............TITPSATTNQPVSFRSF..................SSPSASPTLSTAQLHPQPRQKQPSTPPLSA
DrHCN4L  ...............STPPPSSSSLPSTITTTVTTTTSSITESSVYHHRPIISHGSKDFSVAQLHSQPQLSQASFSSIAA

                   910                                920       930       940       950
HsHCN4   .........FGHFHKALG.GS...............LSSSDSPLLTPLQPGARSPQAAQPSP.....APPGARGGLGLPE
DrHCN4   RLQAAGAHPPGILTTASSNTSA..................LSAGLSTHSPTHSYPPPKHPQTGSLQFASGGGKSGLTLFH
DrHCN4L  S.......LPGFFQQGAAGGEAVHLAVPPASTLTPLIAHSVSPILTQLHATHSNPQPTKPIQDL.......NKAGRTAPQ

            960       970       980       990       1000                1010      1020
HsHCN4   HFLPPPPSSRSPSSSPGQLGQPPGELSLGLATGPLSTPETPP...RQPE........PP...SLVAGASGGASPVGFTPR
DrHCN4   S...PP..PGSPIS.....IHS.........QAPESS....TSSQTQPSFSSYLALERSALASIAQYGSANASPS.YT.P
DrHCN4L  S...PV..TESPVHSKSILEQV.........QSPTSVQSQHPQIGIQPDVLSQLAQEPSALASLAQYGSGNASPC.ST.P

               1030      1040      1050      1060      1070      1080      1090
HsHCN4   GGLSPPGHSPGPPR...TFPSAPPRASGSHGSLLLPPASSPPPPQVPQRRGTPPLTPGRLTQDLKLISASQPALPQDGAQ
DrHCN4   LALSPTVQSPVTGRT..FQYSDPSSAAGSHTSLLLPQSSCPGQLPGHHSSG.TDSPLGRFYEDLNILSSSHPSLGVE...
DrHCN4L  SAWSPTCQSPTAEKMRMSAHSQPSSISGSQTSLLTPQTLFPPPPQPRERGG.SLV..DLPSQDLSTLSMSLPIPGLAEKS

       1100      1110      1120      1130      1140      1150      1160      1170
HsHCN4   TLRRASPHSSGESMAAFPLFPRAGGGSGGSGSSGGLGPPGRPYGAIPGQHVTLPRKTSSGSLPPPLSLFGARATSSGGPP
DrHCN4   ...GAGQ.................SSPGYLSPYLSPTLTPKPCSSVSG.HMSSPVQTSPGPRGQ........ALSVGTSP
DrHCN4L  RAVGRGPQPP.......DLSHHRGSTSG.YSPLPSPPPLVNPFSSIPG.HVVLSRQTSKLSITQ........TGSSGGSP

       1180      1190      1200
HsHCN4   LTAGPQREPGARPEPVRSKLPSNL
DrHCN4   ALSLPR.TSDGDLEPLRSKLPSNL
DrHCN4L  AHGSSD.RSTPGTPSLHPKLPSNL
```

Figure S1
